# Transcriptome data reveals the conservation genetics of *Cypripedium forrestii*, a plant species with extremely small populations endemic to Yunnan, China

**DOI:** 10.3389/fpls.2024.1303625

**Published:** 2024-01-31

**Authors:** Liewen Lin, Lei Cai, Hua Huang, Shengping Ming, Weibang Sun

**Affiliations:** ^1^ Yunnan Key Laboratory for Integrative Conservation of Plant Species with Extremely Small Populations, Kunming Institute of Botany, Chinese Academy of Sciences, Kunming, Yunnan, China; ^2^ Key Laboratory for Plant Diversity and Biogeography of East Asia, Kunming Institute of Botany, Chinese Academy of Sciences, Kunming, Yunnan, China; ^3^ School of Life Science, University of Chinese Academy of Sciences, Beijing, China; ^4^ Lijiang Alpine Botanic Garden/ Kunming Botanical Garden, Kunming Institute of Botany, Chinese Academy of Sciences, Kunming, Yunnan, China

**Keywords:** *Cypripedium forrestii*, conservation genetics, RNA, plant species with extremely small populations (PSESP), orchid

## Abstract

The *Cypripedium forrestii* is an orchid species with extremely small populations (PSESP) in Yunnan, China. *C. forrestii* is range-restricted and less-studied than many orchid species, and it is exposed to various threats to its survival. We investigated its potential habitats and collected 52 samples from eight locations, as well as two outgroup species for reference. We developed genetic markers (SNPs) for *C. forrestii* based on transcriptome sequencing (RNA-seq) data, and analyzed the genetic diversity, population structure, gene flow and demographic history of *C. forrestii* in detail. *C. forrestii* is a taxonomically independent species to protect. We found that the genetic diversity of *C. forrestii* was very low (1.7e^-4^) compared with other endangered species. We identified three genetic clusters, and several populations with distinct genetic backgrounds. Most genetic diversity was found within sampling sites (87.87%) and genetic clusters (91.39%). Gene flow has been greatly limited over the most recent generations, probably due to geographical distance, historical climate change and habitat fragmentation. We also detected a severe bottleneck event brought about by the recent population constraints. These factors, together with its reproductive characteristics, contribute to the population fragmentation and low genetic diversity of *C. forrestii*. Based on our findings, we suggest an integrative conservation strategy to protect and recover the genetic diversity of *C. forrestii* and a further comprehensive study of its ecological traits in the future.

## 1 Introduction

Species with extremely small populations may have difficulties in reproduction and regeneration (Kéry et al., 2000), as well as higher chances of extinction in a single incidental event (Ralls et al., 2018). Small and fragmented populations shaped by bottleneck events may also suffer aggravated inbreeding and the accumulation of deleterious mutations (Ma et al., 2022b). Moreover, species with small populations and/or restricted distributions may also have limited genetic diversity. The loss of genetic diversity due to genetic drift may jeopardize species’ evolutionary potential and lead to outbreeding depression between populations (Spielman et al., 2004; Bijlsma and Loeschcke, 2012). Therefore, the protection of plant species with extremely small populations (PSESP) is urgent, and vital for the conservation of biodiversity (Ma et al., 2013; Sun et al., 2019a). The PSESP concept, which was initiated in Yunnan, China, proposes that emergency conservation of a species requires multidisciplinary study of the species in question, as well as concrete actions by different stakeholders (Sun et al., 2019a; Sun et al., 2019b). The PSESP project promotes initial field investigations and consultation with stakeholders, in order to provide guidelines to prioritize the conservation needs of various plant species, concentrating resources on the most desperate cases (Volis, 2016; Yang et al., 2020). By utilizing and coordinating the strength of government agents, researchers, botanic gardens, local residents and NGOs, the PSESP rescue plan (2010–2020) has achieved a number of conservation objectives (Crane, 2020; Cogoni et al., 2021).

*Cypripedium* L. (Orchidaceae), known as the lady’s slipper orchids, is a terrestrial orchid genus with more than 50 species, primarily distributed in the temperate regions of the Northern Hemisphere (Cribb, 1997). More than half of the known species in this genus, 32 species, are native to China (Cribb, 1997; Chen, 2013), and all of the Chinese species of *Cypripedium* (except the relatively common *C. plectrochilum*) are now one of the National Key Protected Wild Plants of China. China has several endemic species of lady’s slipper orchids, and these species tend to have limited distributions and small population sizes (Cribb, 1997; Chen, 2013). Currently, four particularly rare *Cypripedium* species are considered as PSESP for priority protection (Sun, 2021). The distribution patterns and population genetics of *Cypripedium* orchids are, as is the case for many orchids, limited by their relatively short seed dispersal distance (Brzosko et al., 2017; Kotilínek et al., 2020), low fruiting rate (Suetsugu and Fukushima, 2014; Gargiulo et al., 2021) and the heterogeneity of available microhabitats (Diez, 2007; Hollick et al., 2007; Jacquemyn et al., 2012; McCormick et al., 2012). Human activities, including over-collection for folk medicine and horticultural use, habitat destruction and disturbance also contribute to the decline of wild orchid populations (Brundrett, 2007; Brundrett, 2019). Moreover, genetic studies into various *Cypripedium* species suggest that habitat fragmentation may hamper gene flow between populations (Brzosko et al., 2002; Minasiewicz et al., 2018). This may impair potential resilience to environmental change or habitat loss, and may lead to a higher risk of extinction for these species (Brzosko et al., 2002; Izawa et al., 2007; Qian et al., 2014).

**Figure 1:**
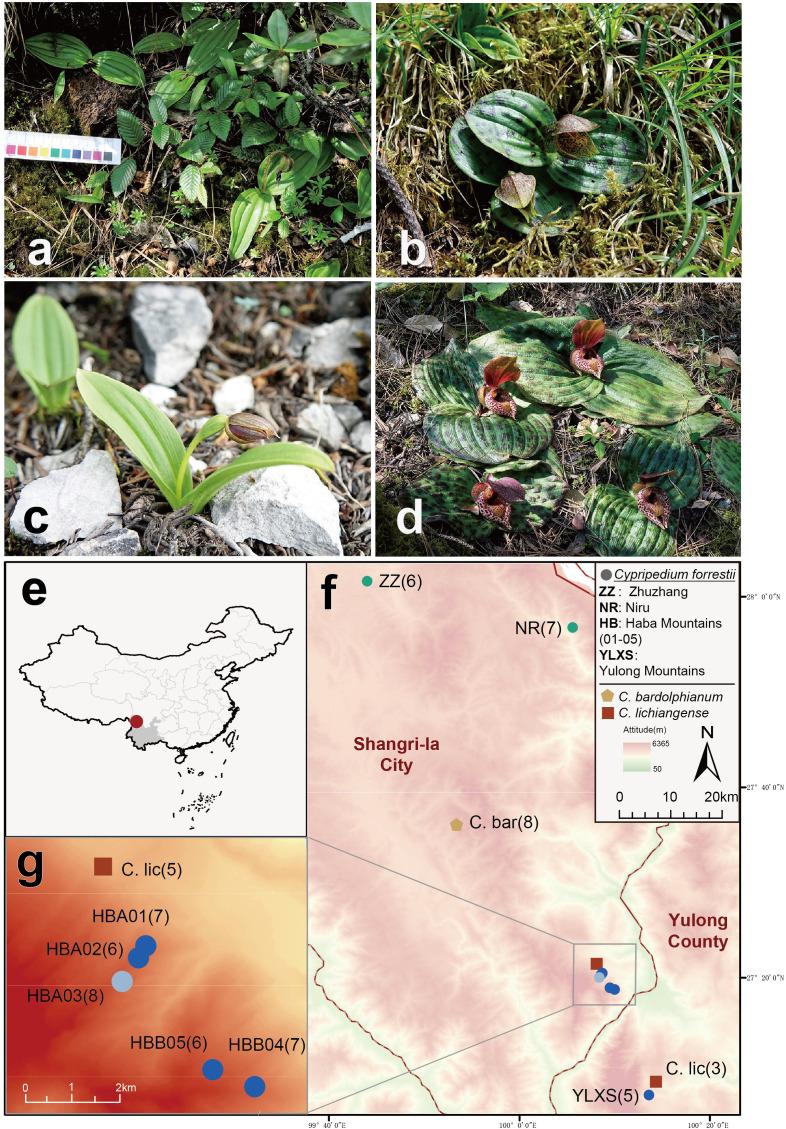
**(A-D)** Photographs of three related *Cypripedium* species. **(A)** *C. forrestii* individuals without spots on the leaves; **(B)** *C. forrestii* individual with spotted leaves; **(C)** *C. bardolphianum*; **(D)** *C. lichiangense*. **(E, F)** Map of sampling sites. **(E)** Position of sampling area in NW Yunnan, China; **(F)** Ten sampling sites of three *Cypripedium* species (*C. forrestii*, *C. bardolphianum* and *C. lichiangense* in Shangri-la and Yulong; **(G)** details of sampling site in the Haba Snow mountains. Numbers in parentheses are the sample sizes at each site. Different colored circles represent three genetic clusters of *C. forrestii* inferred in this study. C.bar and C. lic are the abbreviation of *C. bardolphianum* and *C. lichiangense*, respectively.

Some of the rarest *Cypripedium* species with extremely small populations, including our target species, *C. forrestii* P.J.Cribb (**Figures 1A, B**), have barely received any research attention. Previous studies have focused on its morphology (Cribb, 1997; Chen, 2013) and its phylogenetic position in the genus *Cypripedium* (Li et al., 2011). These studies suggested that its most closely related species are species from Sect. *Trigonopedia*, including the sympatric but relatively broadly distributed *C. bardolphianum* (**Figure 1C**) and *C. lichiangense* (**Figure 1D**). *C. forrestii* can be distinguished by its unique floral traits (Cribb, 1997; Chen, 2013) and leaf polymorphism. Two morphological forms of *C. forrestii* coexist in all populations, one having plain, unspotted green leaves (**Figure 1A**) and one with blackish spotted leaves (**Figure 1B**). As suggested by its Chinese common name, the ‘Yulong slipper orchid’ was first collected on Yulong Snow Mountain (YLXS) in Lijiang, northwest Yunnan, which, for a long time, was the only known population of this species. Our recent field surveys confirmed further populations in the nearby Shangri-la Tibetan Autonomous Prefecture (ZZ, NR, HBA and HBB, see **Figures 1E–G**). *C. forrestii* grows on banks in scrub and at the edge of conifer woodland, at c. 3000-3800 m. It produces small dark-colored flowers in July, which are deceptive to pollinators as no reward (e.g., nectar) is provided. It is a typical clonal plant, and its populations are composed of a series of similar elements, i.e., ramets. Threatened by anthropogenic disturbance, landslides and a low fruiting rate, there are only about 1000 ramets in total. Because of the tillering nature of this plant, there are likely to be fewer than 500 genetically distinct individuals in the wild. *C. forrestii* has been assessed as Critically Endangered by the International Union for Conservation of Nature due to its extremely narrow distribution and small population sizes.

The conservation of *C. forrestii* has been promoted since 2022. Preliminary conservation work has included field investigations, artificial pollination at Lijiang Alpine Botanic Garden, followed up by the germination of seeds and the aseptic culture of seedlings in Kunming Botanical Garden. However, its genetic diversity and population structure are still unknown, and a better understanding of the genetic background is essential for the further study and conservation of this PSESP (Sun et al., 2019a; Yang et al., 2020).

RNA- seq is now a powerful and cost-effective tool to tackle questions in evolutionary history, molecular ecology and conservation genetics (Alvarez et al., 2015; Tyagi et al., 2022), and we utilized transcriptome sequencing data as part of our investigation into the conservation genetics of *C. forrestii*. As reliable reference genome data is lacking for many species in need of conservation, including *Cypripedium* species, a transcriptome generated from RNA-seq data can be an economical and reliable alternative (Alvarez et al., 2015; Ma et al., 2019b). In this study, a full-length transcriptome and genetic marker (SNP) data were developed from RNA-seq data of *C. forrestii* to address the following objectives: (1) to clarify its population genetic structure; (2) to estimate the genetic diversity and genetic differentiation in different populations of this species; (3) to detect any bottlenecks arising from recent population declines; and (4) to evaluate gene flow among isolated habitats and small populations.

## 2 Methods

### 2.1 Sample collection and mRNA preparing

**Figure 2:**
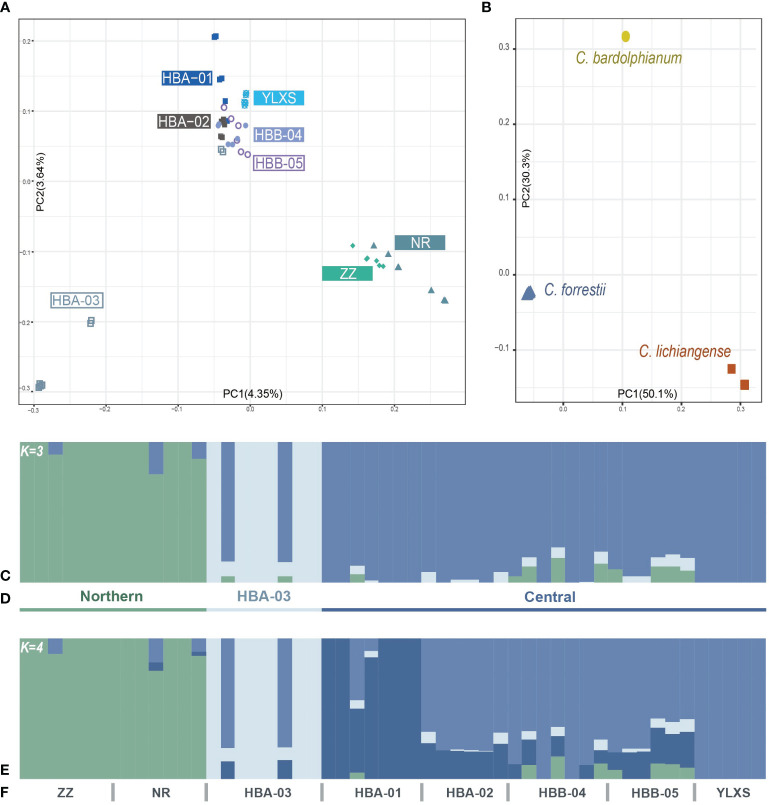
**(A, B)** Population structure of *C. forrestii* inferred by PCA results. **(A)** 52 sampled *C. forrestii* individuals from eight sampling sites; **(B)** *C. forrestii* and its two sister species (*C. bardolphianum* and *C. lichiangense*); **(C-F)** Population structure inferred by Admixture results with **(C)** K = 3 and **(D)** the putative three genetic groups; **(E)** second-best inference when K = 4; **(F)** and the designation of each sampling sites.

Samples of *C. forrestii* were collected from eight locations (**Figures 1E, F**), covering every location in China where this species is known to occur. Geographical coordinates and altitude information were recorded at all sample locations. 5-8 Leaf samples from each *C. forrestii* population were collected in July, the peak growing season for this species. The distance between each sample was at least 10 m to ensure these ramets are genetically independent individuals, as its tillering tolons are mostly 30-50cm long. We avoided unnecessary damage and collected only the minimum amount of leaves from mature individuals to minimize our impact on these rare plants. Only fresh and undamaged leaves were sampled. All leaves were washed and then frozen immediately in liquid nitrogen. In total, 63 samples of *C. forrestii* were sequenced; 34 samples were from individuals with spotted leaves and 29 samples were from non-spotted plants. 11 samples (6 spotted and 5 non-spotted, 1-2 from each location) were used for long-read sequencing and a hybrid transcriptome assembly. 52 samples (28 spotted and 24 non-spotted, 5-8 from each location) were used for NGS sequencing (**Table 1**). Both morphotypes were collected in all locations. We also collected 8 samples from both outgroup species (*C. bardolphianum* and *C. lichiangense*) for further study (**Figure 2**). All outgroup samples were used for NGS sequencing.

**Table 1:** Sampling information and genetic statistics of *C. forrestii* from eight sampling sites and three genetic groups.

Genetic groups	Sampling locations	Number of ramets	Altitude (m)	Number of samples (NGS)	π × 10^4	TajimaD	Ho	He	Fis	Ne(LD)	Ne(HE)	No(CO)
Northern				13	1.80	0.77	0,41	0.37	-0.11	4.3	12.2	3.8
	ZZ NR	50 50	3526 3465	6 7	1.91 1,91	0,58 0,67	0,48 0,48	0,41 0,40	-0.17 -0.18	27.8 3.1	4.9 4.6	5.3 4.5
Central				31	1.72	0,86	0.37	0.33	-0.12	15.5	10.8	5.7
	YLXS HBA-01 HBA-02 HBB-04 HBB-05	50 200 50 200 200	2997 3401 3576 3664 3792	5 7 6 7 6	2.03 1.89 2.02 1.90 1.93	0.69 0.66 0.73 0.48 0.40	0.56 0.50 0.50 0.45 0,44	0,44 0.40 0.42 0.38 0.39	-0.28 -0.26 -0.20 -0.17 -0.12	Infinite 2.2 1.8 Infinite Infinite	2.8 3.1 3.6 4.7 7.2	4.4 4.0 3.4 4.5 4.2
HBA-03	HBA-03	200	3835	8	1.89	0.79	0.53	0.40	-0.32	1.1	2.5	2.4
Overall		1000	–	52	1.71	1.05	0.35	0.32	-0.08(Fit)	16.5	78	2.5

The total RNA of each sample was extracted using RNAprep Pure Plant Kits in accordance with the manufacturer’s instructions (Tiangen, Beijing, China). The mRNA was then purified using mRNA Capture Beads (oligo dT magnetic beads). A NanoDrop 2000 spectrophotometer (Thermo Fisher Scientific, USA) was used to detect RNA purity (A260/280 and A260/230). A Qubit 2.0 Fluorometer (Thermo Fisher Scientific, USA) was used to measure RNA concentration. The integrity of RNA and the presence of DNA contamination were assessed using 1% agarose gel electrophoresis (200V, 10min). Samples were loaded with 400ng according to Nanodrop concentration measurement. Samples for long-read sequencing were pooled together in equimolar ratios, and were sequenced on the Pacific Bioscience RSII platform (Pacific Biosciences, Menlo Parks, CA, USA). The NGS sequencing was performed using a paired-end library with 150bp read length on an Illumina HiSeq 2000 instrument.

### 2.2 Processing of sequencing data

PacBio sequencing data was used for the hybrid assembly of full-length transcriptome. Raw reads were processed in accordance with the Iso-seq workflow recommended by Pacific Biosciences [https://isoseq.how/clustering/high-level-workflow.html, (17 Feb 2023, date last accessed)]. In brief, raw reads were first input into Pbccs v6.4.0 to generate consensus sequences (CCS). Next, primers were removed and the CCSs were demultiplexed using lima v2.6.0. Isoseq3 v3.8.0 was then used for the removal of poly(a) tails and for the clustering and polishing of the outputted isoforms. We obtained a raw reference transcriptome after completing the above steps.

The NGS reads from one sample (YL03, having the highest alignment rate with the Pacbio data) were used for the transcriptome refinement. Trimmomatic v0.39 (Bolger et al., 2014) was used to remove low-quality reads and adapters of NGS raw reads. FastQC v0.11.9 (Andrews, 2010) was then used to evaluate the quality of the trimmed reads. To obtain a high-quality reference transcriptome, the isoform outputs of PacBio data were further corrected using processed NGS reads in Pilon v1.24 (Walker et al., 2014) with the default parameters. After that, CD-HIT v4.8.1 was used to remove redundant isoforms (parameters: -c 0.99 -aS 0.99 -g 1 -d 0 -G 0). To minimize noise from contamination, sequences highly similar to rRNA sequences were removed (inclusion threshold of e-value < 0.01). rRNA sequences were download from the SILVA database (https://www.arb-silva.de). The viral, bacterial and fungal sequences were removed using the standard pipeline of DecontaMiner v1.4 (Sangiovanni et al., 2019) and its pre-built database (based on NCBI database, see https://github.com/amarinderthind/decontaminer). Finally, we obtained the first high-quality full-length transcriptome of *C. forrestii*, which we could then use as the reference sequence for this study.

### 2.3 SNP calling and filtering

Short read NGS data were used for SNP calling. We followed the GATK Best Practices Workflow to identify the SNPs in our RNAseq data [https://gatk.broadinstitute.org/hc/en-us/articles/360035531192-RNAseq-short-variant-discovery-SNPs-Indels-, (17 Feb 2023, date last accessed)]. First, trimmed NGS reads from each sample were mapped to reference sequences using the two-pass mode in STAR v 2.7.10b (Dobin et al., 2013). SAMBAMBA v0.6.6 (Tarasov et al., 2015) was used to filter out reads with mapping quality < 30, and then to sort and index the BAM files. A series of tools in GATK v4.1.9.0 were then applied to the raw mapped reads. GATK MarkDuplicates (Picard) was used to exclude duplicates and GATK SplitNCigarReads was employed to reformat alignments, after which we called SNPs using the default ploidy setting of GATK HaplotypeCaller, since *C. forrestii* and its sister species are diploid (unpublished data). The intermediate GVCFs generated by the ERC mode of HaplotypeCaller were then input into the GATK GenotypeGVCFs for joint calling. GATK SelectVariants was used to select raw SNPs and to remove indels from the joint VCF.

We then filtered the raw SNPs. Since it is not a model organism, we adopted the hard-filtering setting of the VariantFiltration tool as recommended by GATK [https://gatk.broadinstitute.org/hc/en-us/articles/360035890471, (17 Feb 2023, date last accessed)]: –filter-expression “QD < 2.0 || MQ < 40.0 || FS > 30.0 || SOR > 3.0”. SNPs with >20% missing rate and minor allele frequency >0.05 were also excluded. We called the remaining data SNP Dataset1. In addition, we repeated the above-mentioned mapping, SNP calling and hard filtering procedures with the 16 outgroup samples added to the 52 C*. forrestii* samples, and obtained Dataset O.

As unlinked SNPs are required in some of the downstream analysis (e.g., ADMIXTURE and PCA), cluster SNPs were filtered out using GATK VariantFiltration (parameter: -cluster 3 -window 10). We also filtered out potentially linked SNPs with PLINK v1.90b6.21 (Purcell et al., 2007) (parameter: –indep-pairwise 50 5 0.5). Moreover, VCFtools v0.1.16 (Danecek et al., 2011) was used to retain only bi-allelic sites. SNP Dataset2 was thus derived from Dataset1. We also extracted the synonymous SNPs of four-fold degenerate third-codon transversion sites (4Dtv sites) from Dataset1 with the getCdsPep function in ReSeqTools (He et al., 2013). The output, Dataset 3, was used for the analysis of demographic history.

### 2.4 Population structure and genetic diversity

Population structure was inferred using Principal Components Analysis (PCA) and ancestral population estimation based on Dataset 2. PCA was performed in PLINK v1.90b6.21 (Purcell et al., 2007) with the default parameters. The same method was employed to examine potential differentiation between spotted and non-spotted individuals. Ancestral population estimation was performed using ADMIXTURE v1.3.0 (Alexander et al., 2009). The optimal number of ancestral populations (K) was determined by the lowest cross-validation error of K values between 1 and 8. The results from the ADMIXTURE analysis were visualized in pophelper [http://pophelper.com/, (17 Feb 2023, date last accessed)].

The average nucleotide diversity (π) and Tajima’s D value per site were calculated for Dataset 1 and Dataset O, using VCFtools v0.1.16 (Danecek et al., 2011) with non-overlapping 10000bp windows. Heterozygosity indexes (*H_O_* and *H_E_*), pairwise genetic differentiation (*F_ST_*) and inbreeding coefficient (*F_IS_*) of each sampling location and genetic group were calculated in Arlequin v3.5.2.2 (Excoffier et al., 2005) using Dataset 2. The same methods were employed to investigate differences between spotted and non-spotted individuals. An AMOVA analysis was also conducted in Arlequin to assess the genetic variance within and among sampling sites.

NeESTIMATOR v.2.1 (Do et al., 2014) was used to estimate the contemporary effective population size (*N_e_*) using Dataset 2. The heterozygote excess method (HE), the molecular coancestry method (CO) and the linkage disequilibrium method (LD) were all employed. The cutoff was set based on the minor allele frequency within the following interval: 1/(2n) ≤ PCRIT ≤ 1/n, where n is the number of samples (Waples and Do, 2010). Demographic trajectories of *C. forrestii* were inferred from Dataset 3 using Stairway Plot2 v2.1.1 (Liu and Fu, 2020). We estimated the mutation rate to be about 3.9 × 10^-9^ per site per year. The generation time was set as 3 years. The site frequency spectrum (SFS) was inferred from SNP data using the realsfs program implemented in ANGSD v0.938 (Korneliussen et al., 2014). The program returned no valid results when *C. bardolphianum* or *C. lichiangense* was used to infer the ancestral state of SFS, maybe because of the loss of low frequent polymorphism sites. We therefore calculated the folded SFS of all *C. forrestii* samples, since the ancestral state may not be correctly designated without an outgroup reference.

### 2.5 Evaluation of gene flow

The R package “ade4” was employed to test for the presence of isolation-by-distance (IBD) among sampling sites. Recent gene flow among sampling sites was evaluated with a Bayesian inference approach, using BayesAss (Wilson and Rannala, 2003) in BA3SNP v3.0.4 (Mussmann et al., 2019) based on Dataset 2, with 1 × 10^7^ iterations, a burn-in of 1 × 10^6^ steps and a sampling frequency of 1,000. We fine-tuned the two parameters (-a and -f) to adjust acceptance rates for allele frequencies and inbreeding coefficients according to the official user manual (Mussmann et al., 2019).

Since results from BayesAss only represent gene flow among the most recent generations (the last 1–3 generations), Dataset 2 was also analyzed using TreeMix v. 1.12 (Pickrell and Pritchard, 2012). TreeMix requires an outgroup population setting. First, we rooted the graph with the outgroup species using dataset O. We then inferred the gene flow of *C. forrestii*, using its basal lineage suggested by the previous graphs. We constructed graphs of 0–10 migration edges (m), the Optimal m values and graphs were inferred using the *ad hoc* statistic (Δm) and residuals were computed with OptM (Fitak, 2021).

## 3 Results

### 3.1 Sequencing and SNP calling

PacBio sequencing generated 29,383,943 subreads with a mean length of 1,438 bp and a mean quality of 40.1. The raw data of PacBio sequencing was used for a hybrid transcriptome assembly. The CCS process produced 402,442 reads with a mean length of 1,502 bp. After removing primers, demultiplexing, and poly(A) tail removal, 348,844 high-quality reads were used as input for Isoseq clustering, resulting in 32,297 reads. Two passes of error correction were conducted using the NGS sample with the highest alignment rate. The corrected reads had a mean length of 1,576 bp. After subsequently removing redundant isoforms, 15,553 transcripts remained. Any potentially contaminating sequences from rRNA and microbial genomes were then removed. The final reference transcriptome comprised 15,363 transcripts with a mean length of 1,662 bp.

The NGS sequencing of 52 C*. forrestii* samples generated an average of 62.8 million raw reads per sample. The length of the raw reads was 150 bp, and the average Q20, Q30 and GC ratios were 98.5%, 95.0% and 47.9%, respectively. The mean Phred score of nucleotides was 37. After the trimming process, an average of 29.2 million clean reads with a mean length of 135 bp were retained for each sample.

The clean NGS reads were mapped to this reference transcriptome. The mapping rates ranged from 83.87% to 89.49%, with an average of 86.35%. GATK identified 176,657 raw SNPs. After the hard-filtering using GATK, 44,970 SNPs were retained in Dataset 1. The mean depth per site averaged across all individuals was 118. The second round of filtering generated 20,973 bi-allelic and unlinked SNPs, which formed Dataset 2. After extraction of the 4Dtv sites from Dataset 1, a total of 7,876 SNPs were obtained for Dataset 3. Dataset O comprised 245,346 filtered SNPs.

### 3.2 Population structure and genetic diversity

The results of the PCA (**Figure 2A**) indicate that samples from the northern sites, ZZ and NR, form a distinct group (the Northern genetic cluster). Most samples from HBA-03 are also strongly separated from samples from other sites (the HBA-03 genetic cluster). The remaining samples from the Haba and Yulong Mountains (HBA-01, 02, B04, 05, YLXS), which form the central part of the *C. forrestii* distribution area, are more closely related to each other and clustered together in one group (the Central genetic cluster). ADMIXTURE (**Figures 2C, E**) results also support that the optimal number of ancestral populations (K) is 3 (**Supplementary Figure S1A**), and demonstrate that most of the samples from HBA-03 and the two northern sites represented rather “unmixed” genetic backgrounds. The second-best estimation of K is 4 (**Supplementary Figure S1A**). When K=4 (**Figure 2E**), HBA-01 is differentiated from the other central populations, and samples from HBA-01 also stood apart from the other central samples in the PCA graph.

The fixation index values (*F_ST_*) also suggest that samples from the northern sites and from HBA-03 are genetically distinctive from other populations (**Table 2**), as the highest *F_ST_* is found between ZZ and YLXS (0.183), follow by that between HBA-03 and YLXS (0.182). However, the *F_ST_* values between YLXS and other populations are relatively high, even within the central genetic cluster (0.103 to 0.157 when compared with other central sites). Moreover, YLXS shows no genetic mixture with other genetic clusters in the ADMIXTURE clustering analysis, which suggests that YLXS has a unique genetic background. Meanwhile, other samples from the central group are relatively similar genetically, with pairwise *F_ST_* values ranging from 0.029 to 0.096. The lowest pairwise *F_ST_* is found between HBB-04 and HBB-05 (0.029), and the second lowest is between HBA-02 and HBB-05.

**Table 2:** Pairwise *F_ST_* of *C. forrestii* between eight sampling sites.

	ZZ	NR	HBA-03	HBA-01	HBA-02	HBB-04	HBB-05	YLXS
ZZ NR	- 0.122	-						
HBA-03	0.172	0.171	–					
HBA-01 HBA-02 HBB-04 HBB-05 YLXS	0.157 0.137 0.122 0.110 0.183	0.142 0.124 0.118 0.105 0.173	0.145 0.115 0.116 0.097 0.182	- 0.086 0.096 0.065 0.157	- 0.062 0.039 0.127	- 0.029 0.120	- 0.103	-

The individuals with spotted leaves are not significantly separated from those with non-spotted leaves in the PCA (**Supplementary Figure S2**). Pairwise *F_ST_* between spotted and non-spotted individuals is 0.00379, much lower than the pairwise *F_ST_* values between any of the sampling sites (**Supplementary Table S2**), demonstrating that the two leaf polymorphisms have many fewer genetic differences between them than any pair of sampling sites. AMOVA analysis reveal that the vast majority of genetic variation in *C. forrestii* is distributed within 8 sampling sites (87.87%) and within 3 genetic clusters (91.39%) (**Supplementary Table S3**).

PCA, ADMIXTURE and *F_ST_* are also calculated based on Dataset O. The *F_ST_* values between *C. forrestii* and *C. bardolphianum*, and *C. forrestii* and *C. lichiangense* are 0.91 and 0.86, respectively (**Supplementary Table S1**). Each species is distinct and well-separated in the PCA (**Figure 2B**) and ADMIXTURE analyses. ADMIXTURE results do not reveal notable genetic mixture among the three species (**Supplementary Figures S1C, D**).

The nucleotide diversity (π) of *C. forrestii* is 1.71e^-4^ (**Table 1**). At the sampling site level, the diversity values are roughly similar. YLXS has the highest value (2.03 e^-4^), follow by HBA-02 (2.02e^-4^), whereas the HBA-03 and HBA-01 have the lowest π values (1.89 e^-4^). By comparison, π values for *C. bardolphianum* and *C. lichiangense* calculated from Dataset O are 2.10 e^-4^ and 4.36 e^-4^, respectively (**Supplementary Table S4**).

The Tajima’s D values at the site level range from 0.40 (HBB-05) to 0.79 (HBA-03), with an overall average of 1.05 (**Table 1**). The inbreeding coefficient among all sites, *F_IT_*, is -0.08. The inbreeding coefficients within each site, *F_IS_*, are all negative. The smallest value is found at HBA-03 (-0.32), while HBB-05 has the largest (-0.12). This is consistent with the estimation of contemporary effective population size (*N_e_*, **Table 1**), i.e., HBA-03 has the smallest *N_e_* of all the sampling sites. The *N_e_* values vary greatly among the three methods used (HE, LD, CO, as mentioned in the methods section). The results of the LD method may be unreliable in this study, as the confidence intervals of several *N_e_* values are outputted as “infinite”.

Stairway Plot2 reveals a significant drop in effective population size that happened about 1k years ago, and a second decline in the central group about 250 years ago (**Supplementary Figure S5**).

### 3.3 Limitation of gene flow

**Figure 3:**
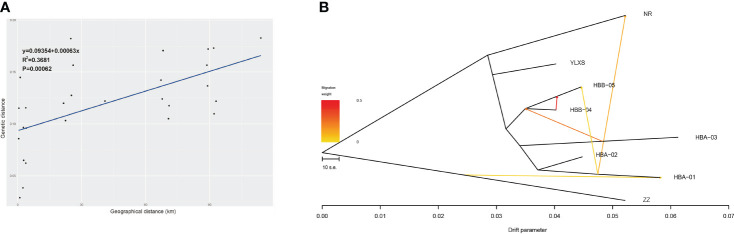
**(A)** Correlation analysis of geographic distance and genetic distance of eight sampling sites of *C. forrestii* (Mantel test). **(B)** Gene flow evaluated by TreeMix.

The Mantel test of IBD suggests that the divergence and gene flow of *C. forrestii* should be significantly limited by geographical distance (R^2^ = 0.37, P < 0.001, **Figure 3A**). However, geographical distance should not be the only constraint. As mentioned above, *F_ST_* values between HBA-03 and other central sampling sites are relatively high, although some samples are collected only a few hundred meters away from each other. Some sites, such as HBA-03 and YLXS, should have lower gene flow to the others, because of the relatively high genetic distances between adjacent sites.

BayesAss analysis of recent gene flow (**Supplementary Table S5**) indicates that gene flow among most sampling sites should be scarce when standard errors are taken into account. Plausible gene flow is likely to occur within the central genetic clusters, especially between HBB-04 and HBB-05 (0.0471 and 0.0541). Moreover, HBB-05 seem to be a major sink for gene flow from nearby sites.

The NJ trees of Treemix suggest that ZZ (from the Northern cluster) is the basal lineage of *C. forrestii* when the root is set as *C. bardolphianum* or *C. lichiangense*. No gene flow is detected between these sister species (**Supplementary Figure S3**). When the outgroup species were included, Treemix fails to predict additional migration events within *C. forrestii* (except between NR and HBA-01). This could be due to the smaller intra-species differences, which are less detectable compared to the inter-species differences. Next, ZZ is set as the root to calculate the gene flow, resulting in a fairly stable topology. The number of predicted migration edges in TreeMix is 5 (**Supplementary Table S6**; **Supplementary Figure S4**), as it corresponds to the highest likelihood (Δm score) when m=5. In this model, Treemix suggests that previous gene flow or admixture events occurred among most sampling sites (**Figure 3B**). The strongest migration event occurred from HBB-05 into HBB-04, and migration between HBA-01 and NR was also detected. No gene flow was detected between YLXS and the other sampling sites. Besides the NR and ZZ populations, the HBA01 and 03 populations also had large drift parameters, indicating that they may be genetically well separated from the other populations.

## 4 Discussion

### 4.1 *C. forrestii* is a taxonomically independent species to protect

Recent studies reveal that the extremely small populations of endangered plant species may result from various factors, including over exploitation (Yu et al., 2021; Ma et al., 2022a), deforestation (Liu et al., 2019; Ma et al., 2022b; Yang et al., 2022) as well as from severe bottleneck events due to historical climate change (Ma et al., 2022b; Yang et al., 2022). The uplift of mountains and climate fluctuations may also facilitate inter-species hybridization (Ma et al., 2019b; Yang et al., 2023). Moreover, studies investigating *Primula* (Ma et al., 2019a) and *Cypripedium* (Hu et al., 2011) have suggested that asymmetric gene flow from sympatric aliens may put rare species at increased risk of extinction by decreasing reproductive fitness of parental species and inducing genetic swamp. Our genetic study tentatively rules out the possibility of a hybridization event between *C. forrestii* and *C. bardolphianum*, or between *C. forrestii* and *C. lichiangense*. *C. forrestii* should therefore be studied and protected as an independent species. In addition, the individuals with and without spotted leaves are two morphotypes of one independent species. As suggested by the AMOVA analysis (**Supplementary Table S3**) and the pairwise *F_ST_* values (**Supplementary Table S4**), most RNA-sequence variants occur within populations and genetic clusters, instead of different leaf morphs.

### 4.2 Relatively low genetic diversity compared to other threatened species

The nucleotide diversity of *C. forrestii* (0.17e^-3^ at the species level) is very low. Previous genetic studies of other orchid species data are based on chloroplast DNA fragments, and yielded much higher nucleotide diversity. For example, the nucleotide diversity of *C. japonicum* is 0.88e^-3^ (Han et al., 2022); *C. tibeticum* has a much higher nucleotide diversity of 1.52e^-3^ (Guo et al., 2019). However, nucleotide diversity estimated using transcriptome or genome data should be more accurate, as it was computed based on a much higher number of loci. In comparison to other threatened or endangered species studied with transcriptome data, the nucleotide diversity of *C. forrestii* is still very low. For example, *Pseudotaxus chienii* has a nucleotide diversity of 0.7e^-3^ (Liu et al., 2021). *Cupressus gigantea* and *Cupressus duclouxiana* exhibit nucleotide diversities of 2.9 e^-3^ and 3.1e^-3^, respectively (Ma et al., 2019b). Additionally, the protein coding region of *Acer yangbiense* shows a nucleotide diversity of 1.88e^-3^ [**Supplementary Data** of (Ma et al., 2022b)]. Moreover, the whole species level nucleotide diversity of *Cypripedium forrestii* was still lower than the very few samples of *Cypripedium lichiangense* (0.4e^-3^).

Nucleotide diversity values can be greatly altered by the type of genetic marker used in the analysis, the sequencing technique and the sampling size, and therefore, any direct comparison between species may be arbitrary. Furthermore, nucleotide diversity values and conservation status are not necessarily correlated. However, it is generally believed that a higher genetic diversity should enhance the potential of species to adapt to the ever-changing environment (Zhao et al., 2019; Ma et al., 2021). Therefore, a low nucleotide diversity could be a critical indicator of species that require the most urgent protection. The low nucleotide diversity may reflect highly conserved protein-coding genes and functional elements. However, a better explanation may be that *C. forrestii* accumulates fewer mutations over time. This hypothesis is supported by our field observations and horticultural experience, i.e., the plant mainly reproduces asexually though tillering, and has low rates of flowering and fruiting. Another possible explanation for the low nucleotide diversity could be the loss of polymorphism sites, especially low frequency alleles, due to genetic drift. This will be discussed in the following section.

### 4.3 The bottleneck and declining population of *C. forrestii*

Negative *F_IS_* values are observed at all sampled locations and in all genetic clusters. This phenomenon has also been reported in many other orchid species at the population and/or at the species level, e.g. *Pelatantheria scolopendrifolia* (Yun et al., 2020); *Serapias lingua* (Pellegrino et al., 2015); *Spiranthes spiralis* (Machon et al., 2003); *Cypripedium calceolu*s (Minasiewicz et al., 2018; Gargiulo et al., 2021) and *C. reginae* (Kennedy and Walker, 2007); as well as plants from other families (Stoeckel et al., 2006; Cabrera-Toledo et al., 2008; Yang et al., 2022). The negative inbreeding values indicate the heterozygote excess within the population, which may result from certain kinds of heterozygote advantage during natural selection (Alvarez-Buylla et al., 1996). However, a small sample size may also cause negative inbreeding values (Balloux, 2004). Notably, the sampling site with the lowest *F_IS_* values (HBA-03) has the highest Tajima’s D value and the lowest *N_E_*, and the site with the highest *F_IS_* values (HBB-05) has the lowest Tajima’s D and highest *N_E_* values. Though originally designed to test the neutrality of DNA polymorphism data (Tajima, 1989), Tajima’s D value can be an effective tool in the detection of selective forces and bottleneck events (Simonsen et al., 1995; Nielsen, 2001). The overall Tajima’s D value of 1.05 may suggest the loss of low frequency alleles across the transcriptome, which is a typical consequence of a bottleneck event.

Stairway Plot2 suggests a continuous decline of the population size. *C. forrestii* is a typical alpine plant that favors a cold environment, and may be susceptible to global warming and contemporary climate fluctuations. During our field surveys, we found that most of its growing area was affected by livestock grazing. Increasing human activities in its suitable habitat may also contribute to its recent population decline.

The severe population decline brought by the bottle neck events is one of the initial causes of heterozygote excess, which can be enhanced by plant’s pollination characteristics like dioecism, self-incompatibility and unbalanced sex ratios (Balloux, 2004; Stoeckel et al., 2006; Cabrera-Toledo et al., 2008). Self-pollination can result in the clearance of heterozygote excess, whereas several studies have shown that deceptive pollination promotes cross-pollination in many orchid species (Jersáková et al., 2006). As mentioned, *C. forrestii* produces deceptive flowers, therefore its heterozygote excess should be reinforced by the cross-pollination between different genetic individuals.

### 4.4 The limitation of gene flow

AMOVA analysis reveals that the vast majority of genetic variation is found within populations. *F_IS_* values between some sites are very small, which suggest that individuals from these sites are very similar genetically. It is possible that all sites and genetic clusters used to be a whole entity with extensive genetic connection. The Treemix result suggests that most sites used to be connected, and that there may have been previous long-distance gene flow from the Haba mountains and the northern sites to other sites. This connection, if it ever existed, has been lost, possibly during the most recent population decline and habitat fragmentation. BayesAss analysis suggests that there has been barely any gene flow between most sites in recent generations. We do not find any populations between the Haba mountains and the two northern sites. Also, the hot and dry valley of the Jinsha river separates YLXS from the other populations, and villages, farmland and ranches have been replacing forest and shrubland in the lower altitude areas.

A certain degree of connectivity still remains between HBB-04 and HBB-05, probably due to the short geographical distance between the two sites. However, interestingly, the short geographical distance between HBA-03 and its neighbor does not seem to increase connectivity between these sites. Noticeably, as demonstrated in **Figures 2A** and **C**, two individuals from HBA-03 show a certain degree of genetic mixture with other genetic clusters, while other individuals have a “unmixed” genetic background. This is also observed in some individuals from the northern group and HBB-04 (**Figures 2C, E**). One possible explanation is that, although rather scarce, there is a certain amount of seed flow between the sites. However, this seed flow does not necessarily bring in actual gene flow. As most individuals do not flower annually and the fruiting rate is low, the chance of producing offspring with mixed genetic background is likely to be slim, even given occasional seed flow between populations. In contrast, genetically mixed offsprings seem to be more common in the other central sites. This specie may merely exists and reproduces asexually in the “second-rate” habitats (the northern sites and HBB-03) and is only inclined to flower in particular environments. It is also possible that presence or abundance of pollinators vary greatly in different sites. Further field observations and pollination ecology studies should help answer this question.

### 4.5 Conservative suggestion of *C. forrestii* based on genetic study

According to our field investigation and genetic study, *C. forrestii* is facing four major threats: (1) a very narrow distribution; (2) low genetic diversity; (3) a small population size and (4) the loss of gene flow between most sites. In view of these factors, the upcoming conservation effort should cover these aspects: (a) *in situ* protection; (b) *in vitro* conservation; (c) cultivation and reintroduction; (d)further exploration of other biological and ecological characteristics.

First, the *in situ* protection should be enhanced. All known sites of *C. forrestii* except ZZ are located within protected areas such as national parks or nature reserves. Cooperation between local government and related authorities can play a vital role in the protection of biodiversity in such areas. Practicable measures include setting up infrared cameras, warning signs and forest ranger patrol. One population is located in the Yulong Snow Mountains, which is one of the most famous scenic spots in Yunnan. Public education and tourism regulation should be enhanced in this particular site. On the other hand, the nearby mountains in Sichuan, for example, may comprise habitat suitable for *Cypripedium*, but to date, these mountains have not been thoroughly investigated. Therefore, field investigations in close-lying areas should be conducted to search for new populations.

The site ZZ is not located in any protected area and is facing the strongest disturbance. HBA-01 and HBA-03 comprise healthy numbers of individuals with a unique genetic background. Collection of ramet, pollen and fruit can be considered for these sites. Artificial pollination could be an effective way to enhance the fruit production and genetic diversity of *C. forrestii*. Since the genetic distances between most sites are relatively small, cross-pollination between sites, at least within genetic clusters, should be appropriate. Our study shows that gene flow between the Yulong Snow Mountains and other sites has been cut-off for a long time. Therefore, cross pollination between individuals in the Yulong Snow Mountains and other central genetic clusters (e.g., HBB-04 and 05) should be a priority to restore gene flow and promote genetic diversity.

The Kunming Botanical Garden is currently engaged in the *in vitro* conservation of *C. forrestii*, which includes aseptic cultivation of seedlings. Additionally, *in situ* seed baiting experiments can be conducted to detect mycorrhizal fungi that enhance the germination. These efforts aim to provide a large number of seedlings and establish a fundamental understanding of the species, which will serve as a foundation for future reintroduction work. Site monitoring is also crucial in understanding the relationship between the environment and reproductive fitness. It is important to identify suitable reintroduction sites where the plant can regenerate and thrive successfully.

The pollination ecology and determinants of leaf vaiegation of *C. forrestii* are still not fully understood. Further observation is required to identify the pollinators of this species and determine if there are any reproductive difficulties. Furthermore, its leaf patterns may have implications for photosynthesis and interactions with herbivores. Investigating leaf polymorphism and its impact on the growth and fitness of the species would provide valuable insights for population management strategies.

*C. forrestii* is now included in the PSESP protection project. Preliminary conservation actions, including artificial pollination and the aseptic cultivation of seedlings, are already underway. These initial conservation efforts are crucial for the long-term survival and recovery of this specie. By actively cooperating with various stakeholders and implementing conservation measures, there is hope for the population to stabilize in the future.

## Data availability statement

The datasets presented in this study can be found in online repositories. The names of the repository/repositories and accession number(s) can be found below: BioProject, PRJNA1029356.

## Author contributions

LL: Writing – original draft, Writing – review & editing. LC: Writing – review & editing. HH: Writing – review & editing. SM: Writing – review & editing. WS: Writing – review & editing.
