# Supplementary figures and images for: Transcriptome data reveals the conservation genetics of Cypripedium forrestii, a plant species with extremely small populations endemic to Yunnan, China
Source: Front Plant Sci. 2024 Jan 31;15:1303625. doi: 10.3389/fpls.2024.1303625 (PMC10864665; doi:10.3389/fpls.2024.1303625)

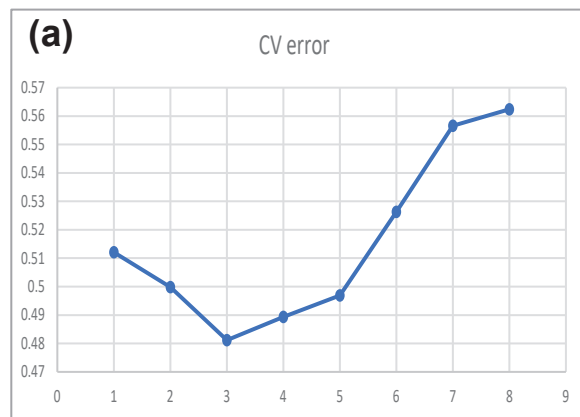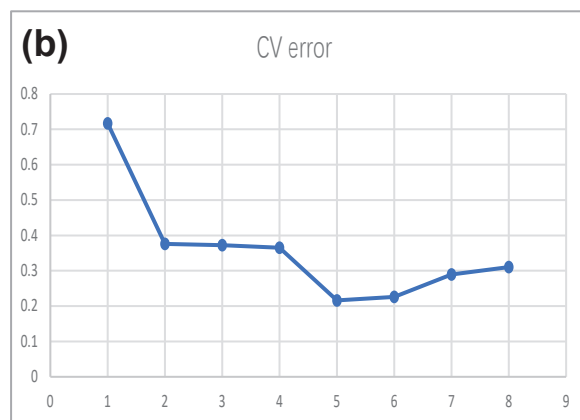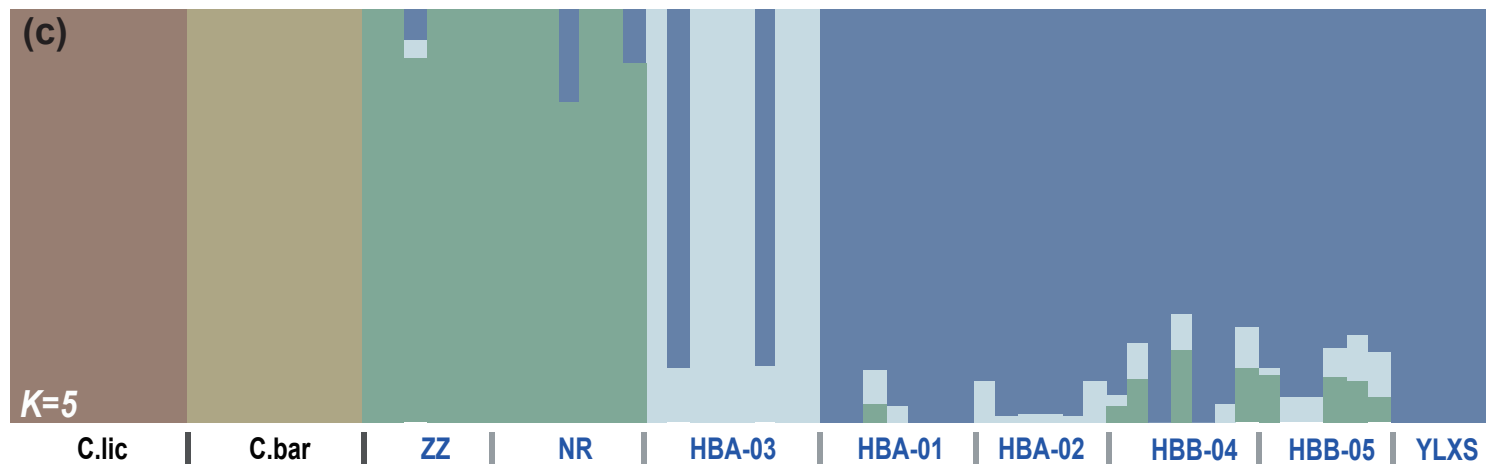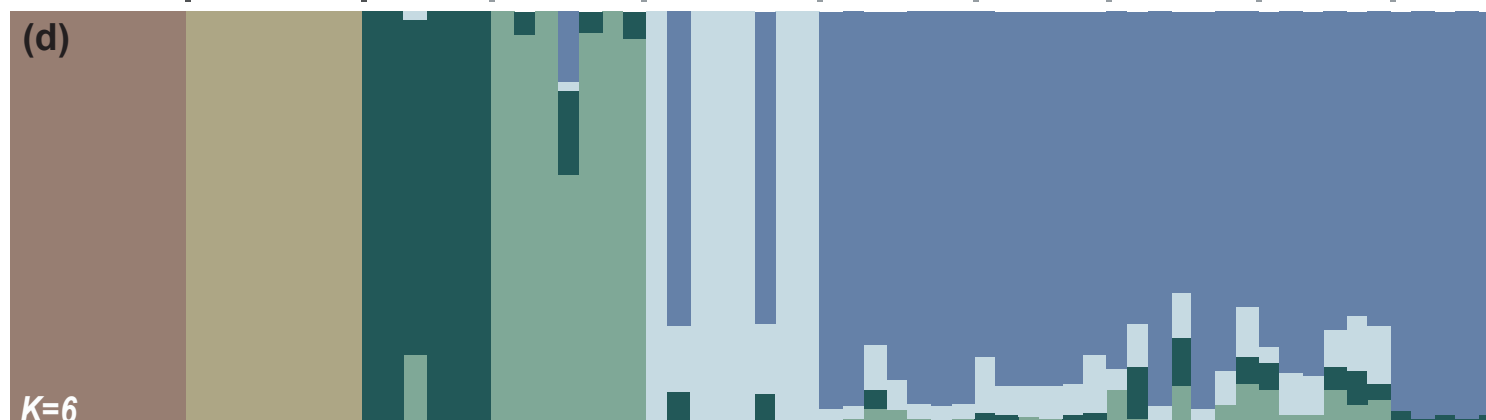

Supplement: Supplementary file 1 [file Image_1.pdf]

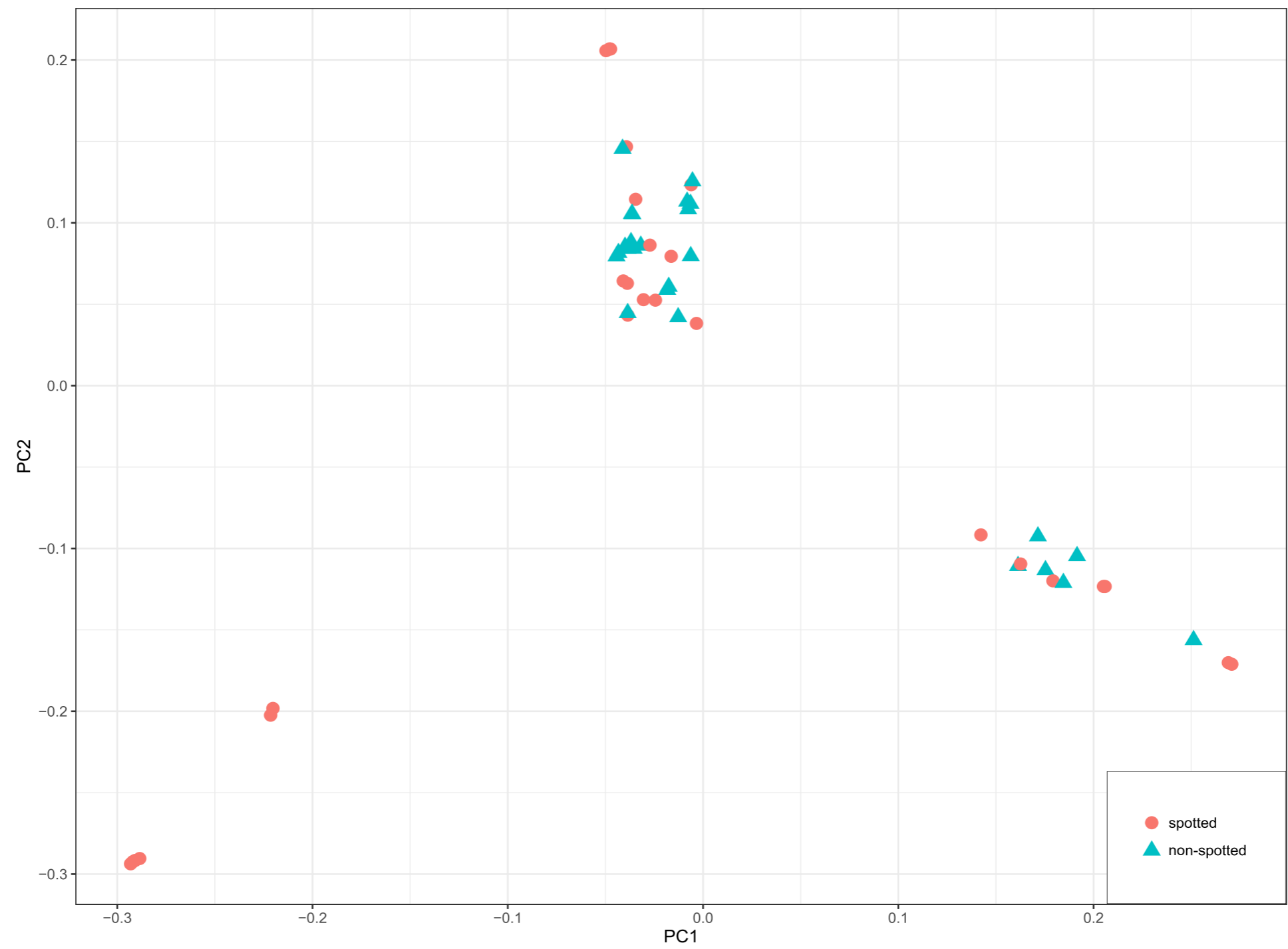

Supplement: Supplementary file 2 [file Image_2.pdf]

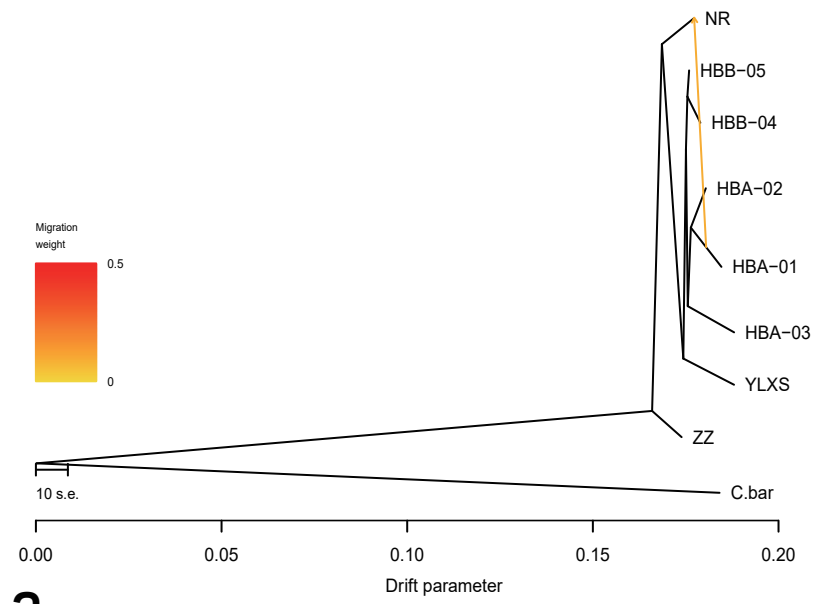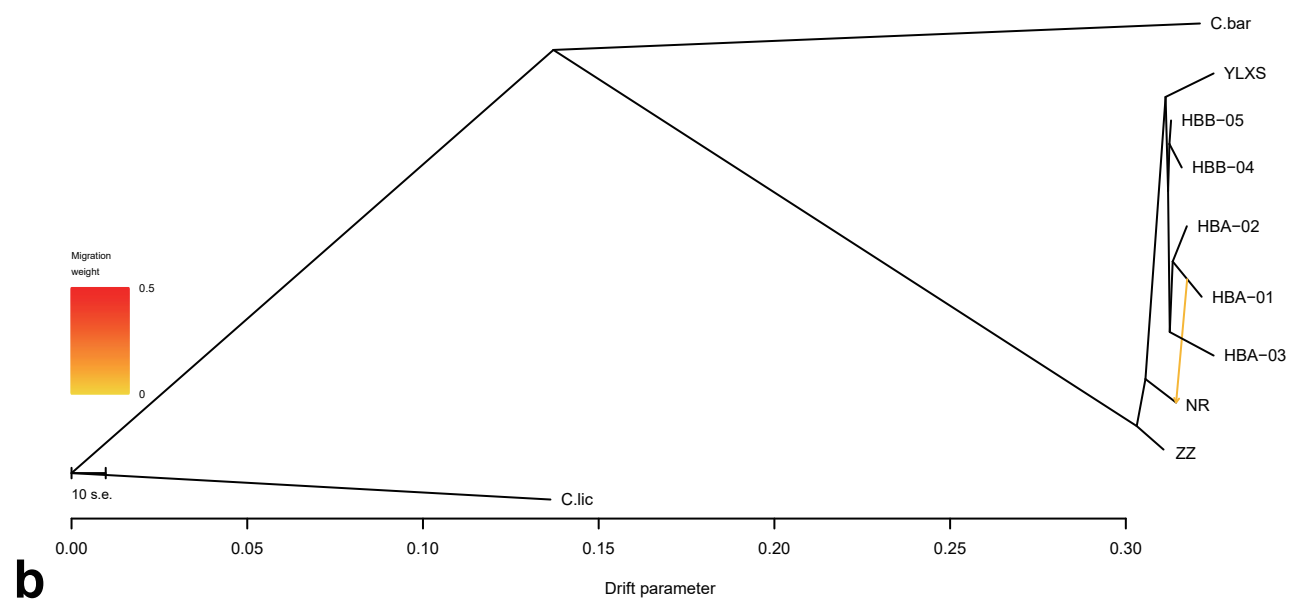

Supplement: Supplementary file 3 [file Image_3.pdf]

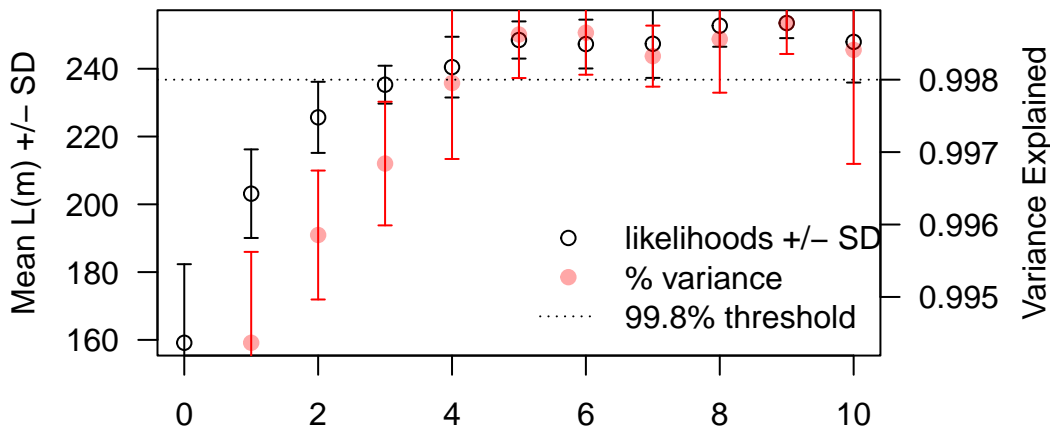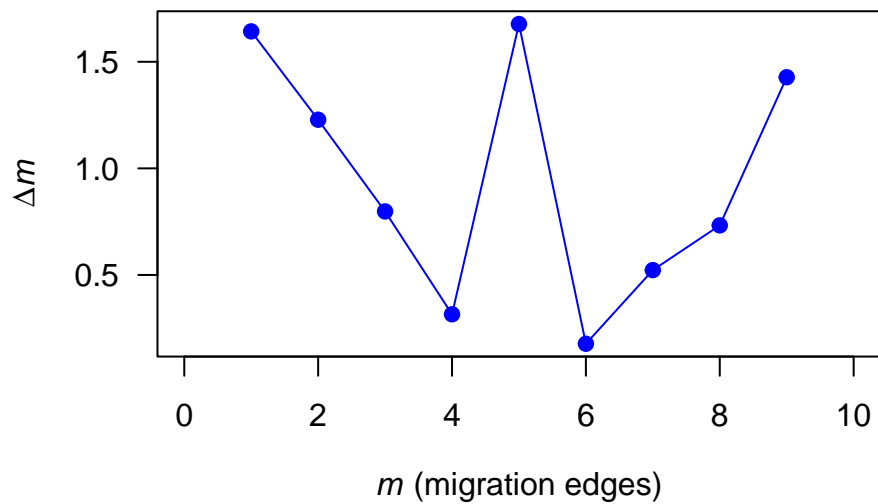

Supplement: Supplementary file 4 [file Image_4.pdf]

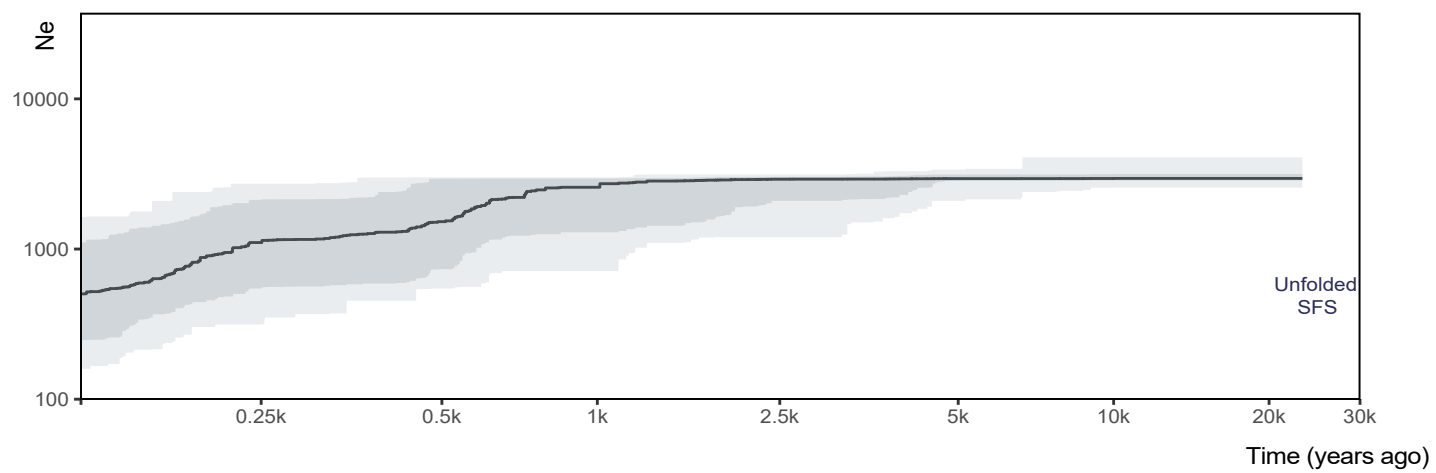

Supplement: Supplementary file 5 [file Image_5.pdf]
